# MeCP2 mediated dysfunction in senescent EPCs

**DOI:** 10.18632/oncotarget.20961

**Published:** 2017-09-16

**Authors:** Chunli Wang, Fei Wang, Zhen Li, Liya Huang, Qing Cao, Shuyan Chen

**Affiliations:** ^1^ Department of Geriatrics, Xinhua Hospital Affiliated to Shanghai Jiao Tong University School of Medicine, Shanghai, China

**Keywords:** EPCs, senescence, MeCP2, SIRT1, Gerotarget

## Abstract

Aging endothelial progenitor cells (EPCs) exhibit functional impairment in terms of proliferation, migration and survival. SIRT1 plays an important role in improving EPCs function. MeCP2, another important epigenetic regulator, is involved in regulating many life-related activities such as cell growth, death and senescence. Here we aim to explore the effect of MeCP2 on the functional activities of senescent EPCs and the underlying mechanisms. By using western blot and real-time PCR, we found that the expression levels of MeCP2 were up-regulated and SIRT1 were down-regulated with replicative senescence and H_2_O_2_-induced senescence. Through transduction with adenoviral vectors, EPCs overexpressing MeCP2 had significantly reduced EPCs function, and silencing MeCP2 improved EPCs function. In addition, the protein and mRNA levels of SIRT1 were decreased with MeCP2 overexpression and increased with MeCP2 knockdown. Through co-transfection of EPCs with MeCP2 and SIRT1, we observed that SIRT1 could reverse the effects of MeCP2 on EPCs. In summary, our work demonstrated that MeCP2 inhibited SIRT1 in senescent EPCs.

## INTRODUCTION

In aging, endothelial dysfunction is a prominent health issue that eventually progresses to atherosclerosis and other vascular diseases [1]. Endothelial progenitor cells (EPCs) are proposed to have the ability to repair injured endothelium cells by replacing damaged or dead endothelial cells [2-4]. Experiments in animal models verified that EPCs can repair impaired endothelial cells and improve angiogenesis and tissue perfusion in ischemia [5, 6]. EPCs transplantation appears to have therapeutic potential, and it needs further laboratory investigations to confirm [7]. Senescent EPCs usually experience function failure [8]. Although there was no correlation between EPCs and individual risk factors, including age, diabetes, hypercholesterolemia and hypertension [9], there was a correlation between the number of EPCs and the combined risk factor score of age and age-related diseases [10]. Thus age and age-related diseases may accelerate and aggravate EPC dysfunction, leading to limitations in the repair capacity of autologous EPCs’ [3, 11-13]. Therefore, it seems crucial to clarify the mechanism underlying aging EPC dysfunction, to develop therapeutic interventions to improve EPC function.

Recent studies have indicated that aging stem cell dysfunction is epigenetic [14]. DNA methylation is an important epigenetic factor. Studies have shown that special CpG islands appeared with hyper-methylated in aging [15, 16]. DNA methylation can repress target gene transcription but it can also act as a signal to inhibit genes by specifically binding methylated CpG protein (called methyl-CpG binding protein). MeCP2 is an important member of this family, it can bind not only methylated DNA but also un-methylated or hemi-methylated DNA, and can then recruit DNA-methyltransferase 1 (DNMT1) to induce DNA methylation and repress transcription [17-19]. Several studies have revealed MeCP2 expression disorder in many types of pathological tissues and cells [20-22], but whether MeCP2 is involved in the regulation of senescent EPC dysfunction is unclear.

SIRT1 (silent information regulator type 1), a nicotinamide adenine dinucleotide (NAD+)-dependent histone deacetylase, has been demonstrated to play a critical role in the regulation of cell survival, replicative senescence, inflammation, and metabolism through the deacetylation of histones and other cellular factors including the transcription factors p53, NF-κB, Ku70, the forkhead transcription factors (FOXOs), and the transcriptional regulator PGC-1α [23]. SIRT1 has been demonstrated to display significant life span extension and delay aging [24-26]. Our previous studies have indicated that SIRT1 protected against H_2_O_2_-induced EPC apoptosis, and SIRT1 exerted its anti-apoptotic effects by inhibiting FOXO3a [27, 28]. We also previously showed that FOXO3a overexpression causes EPC dysfunction through transcriptional inhibition of its downstream target genes such as CDK2, cyclin D1 and PCNA [29, 30]. These results indicate that SIRT1 can improve EPC function.

In this study, based on our previous research results and sufficient reference support, we proposed that MeCP2 could induce the dysfunction of EPCs in aging through repressing the expression of SIRT1. Now we will test this hypothesis.

## RESULTS

### Culture and identification of EPCs

**Figure 1:**
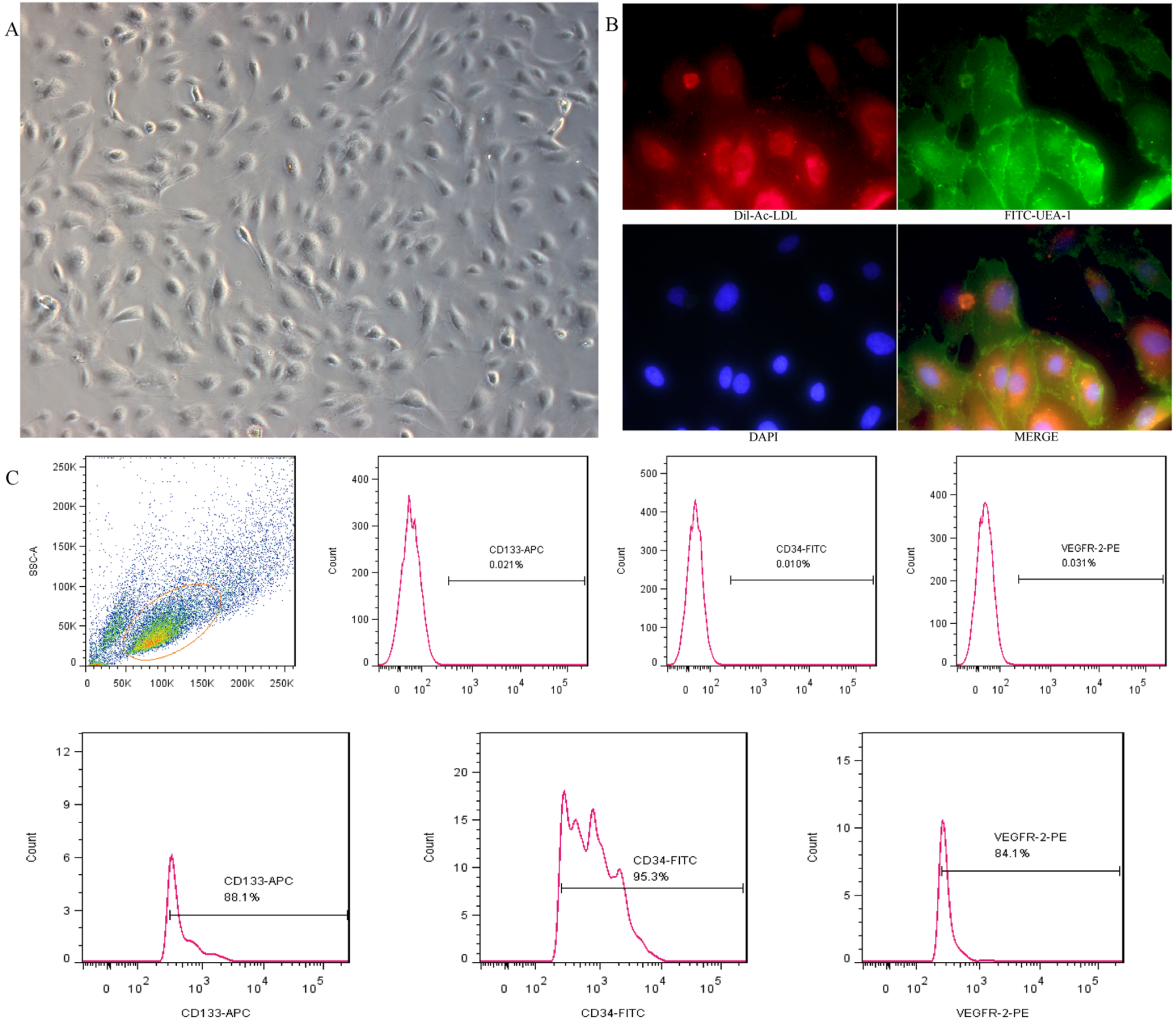
Cultivation and identification of EPCs derived from umbilical cord blood **A.** EPCs exhibited a cobblestone-like cell monolayer at 14 days after seeding(X100). **B.** Uptake of Dil-Ac-LDL and binding of FITC-UEA-1 were observed with a fluorescence microscope (X400). **C.** A representative FSC/SSC plot and the expression of EPC markers (CD34, CD133 and VEGFR-2) analyzed by flow cytometry.

Under the present culture conditions, the adherent cells grew in colonies and showed a cobblestone morphology after two weeks of isolation (Figure 1A), they were double-positive stained for Dil-Ac-LDL and FITC-UEA-I (Figure 1B). Flow cytometry analysis revealed that the positive expression rate was 95.3% for CD34, 88.1% for CD133, and 84.1% for VEGFR-2 (Figure 1C). All these are considered important markers of EPCs.

### Replicative senescence and H_2_O_2_-induced senescence model

**Figure 2:**
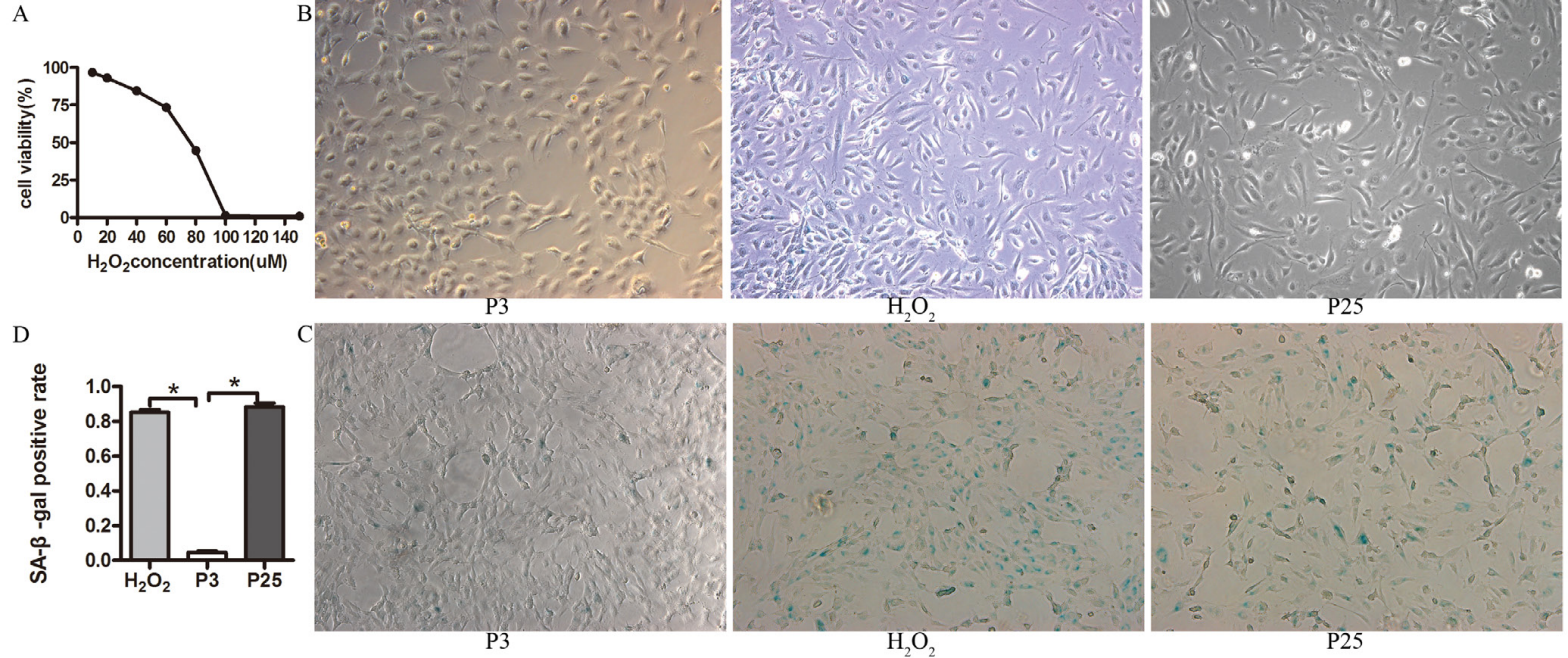
Characteristic of the senescence model **A.** Cell viability with different concentration of H_2_O_2_ determined by CCK-8 assay, EPCs was co-cultured with different concentration of H_2_O_2_ for 24 h, then examined the cell viability by CCK-8. **B.** The morphology of three groups(X100). **C. D.** Beta-gal staining of the three groups (X100) (the results of positive blue stained cell numbers are reported as mean±SD of three independent experiments, *P<0.05 vs. P3).

We verified the senescence model by examining the cells for the morphology of senescence. The third passage (P3) was small in size and had cobblestone-like morphology. Through repeated subculture until the 25th passage (P25), the EPCs appeared large in size and irregular in shape, and some were tree branch-like and polygonal or long spindle-shaped. P3 that was incubated with different concentrations of H_2_O_2_ exhibited variable viability (Figure 2A). P3 that was incubated with 20 µM H_2_O_2_ for 24 h displayed the same morphology as P25 (Figure 2B) when cultured for another 3 days (H_2_O_2_).

**Figure 3:**
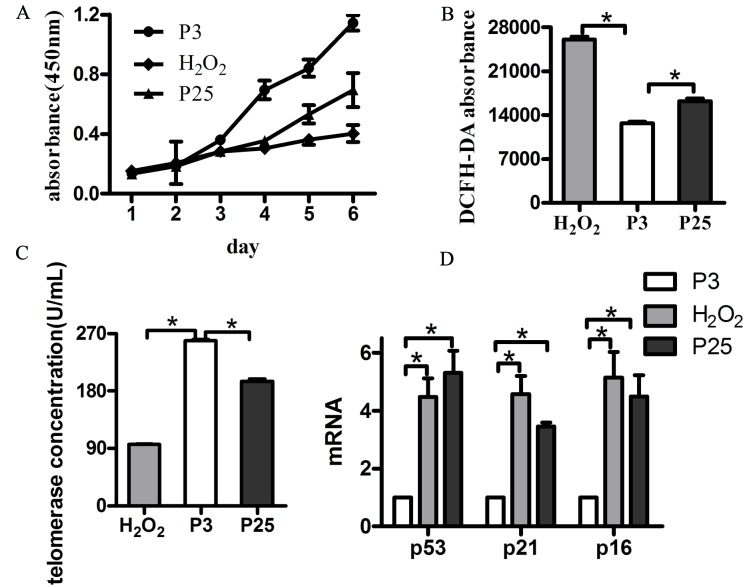
Hallmarks of a senescent model **A.** Growth curve of the three groups (different groups of EPCs were cultured by six consecutive days of testing using the CCK-8 kit). **B.** Reactive oxygen assay using DCFH-DA. **C.** Telomerase activity concentration of the three groups. **D.** Age-related gene mRNA expression. (data were reported as mean±SD, n=3, *P<0.05 vs. P3).

Then, we verified the senescence model by examining the markers and characteristics of senescence. The positive staining rate of P3 was less than that of P25 and H_2_O_2_ by Senescence-associated β-galactosidase (SA-β-Gal) assay (Figure 2C, 2D). P25 and H_2_O_2_ grew more slowly than P3 (Figure 3A). DCFH-DA fluorescence in P3 was less than H_2_O_2_ and P25 (Figure 3B). The TE concentration of the regression equation was as follows, Y=−2.1655+103.0937X ; The TE concentration in P3 was much greater than that in H_2_O_2_ and P25 (Figure 3C). p53, p21 and p16 mRNA levels in P3 were lower than those in H_2_O_2_ and P25 (Figure 3D).

### EPCs function declined with senescence

**Figure 4:**
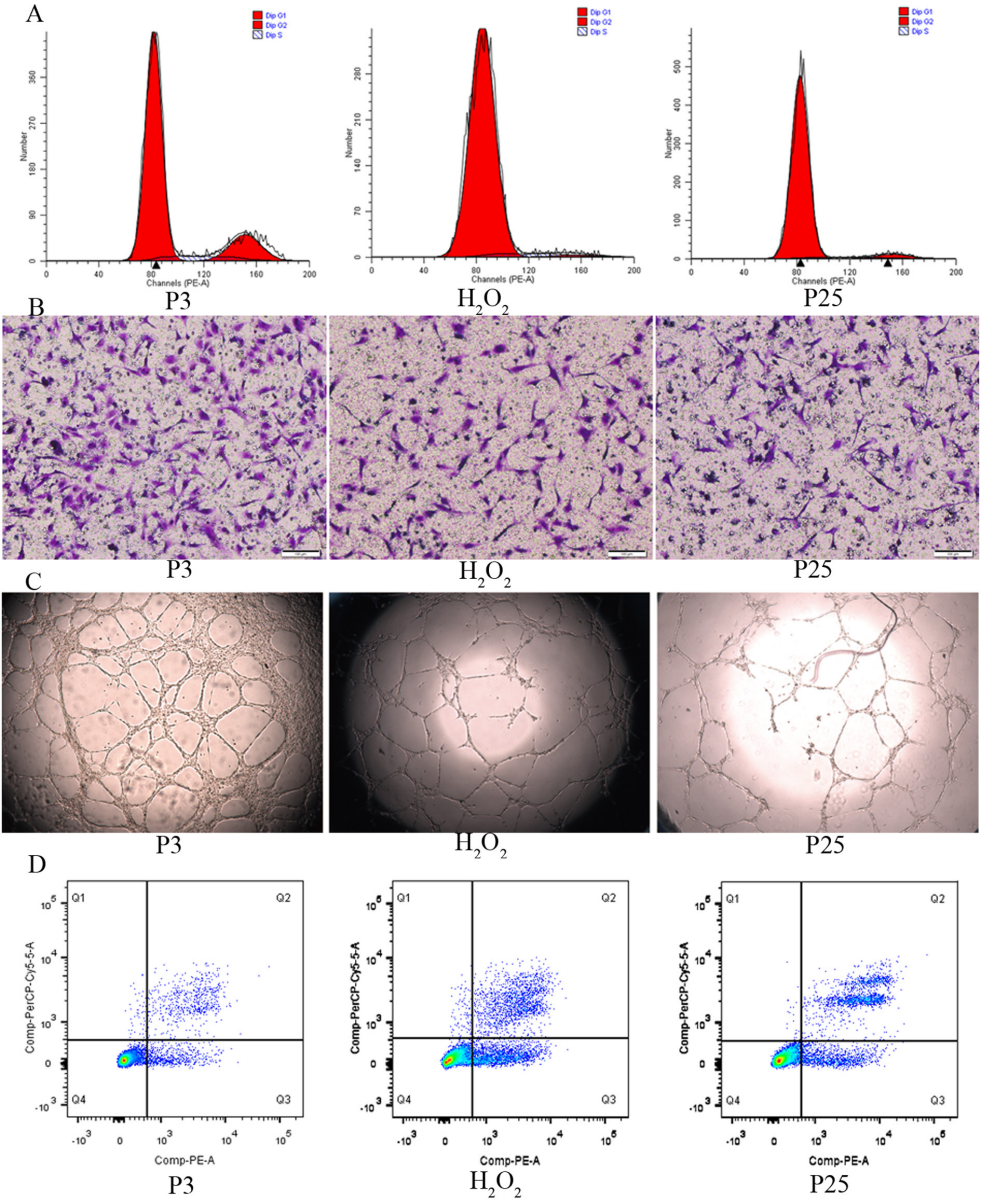
Function comparison of the three groups **A.** Cell cycle by flow cytometry(proliferation index (PI) was used to compare, PI=(S+G2M)/(G0/1+S+G2M)). **B.** Migration ability by transwell assay(X100, the number of dyed purple cells in three randomly microscopic fields were used to access migration ability). **C.** Tube formation of the three groups(X40, total loops were used to evaluate the tube formation ability) **D.** Cell apoptosis by flow cytometry(the percentage of apoptotic cells with Annexin V positive expression was used to assess apoptosis). (three independent experiments were repeated, and the same result were done)

After validating the senescence model, we verified dysfunction in the senescent EPCs. The cell cycle assay is shown in Figure 4A. The proliferation index (PI) that was calculated according to the formula (PI=(S+G2M)/(G0/1+S+G2M)) showed that P3 grew faster than H_2_O_2_ and P25. Cell doubling time showed similar results (Table 1). The amount of cell migration in P3 was greater than in H_2_O_2_ and P25, as shown by Transwell assay (Figure 4B). The tube formation assay is shown in Figure 4C. The tube formation ability of P3 was greater than that of H_2_O_2_ and P25. The cell apoptosis assay revealed that the apoptosis rate of P3 was less than that of H_2_O_2_ and P25 (Figure 4D).

**Table 1:** Three groups of cell doubling time($\bar{x}\pm s$)

group	N_0_(*10^4^,numbers)	N_t_(*10^4^,numbers)	TD(d)
P3	1.70±0.05	9.00±2.65	0.82±0.14
H_2_O_2_	1.20±0.03	2.50±0.50	3.01±1.24^ⱷ^
P25	1.50±0.01	2.17±0.76	2.65±0.81^ⱷ^

TD (Cell doubling time) was calculated according to the formula: $TD=t\frac{\mathrm{lg}2}{\mathrm{lg}N_{t}-\mathrm{lg}N_{0}},t=2$

^ⱷ^*P* = 0.014<0.05 vs. P3, ^ⱷ^*P* = 0.035<0.05 vs. P3.

### MeCP2 and SIRT1 expression with age in EPCs

**Figure 5:**
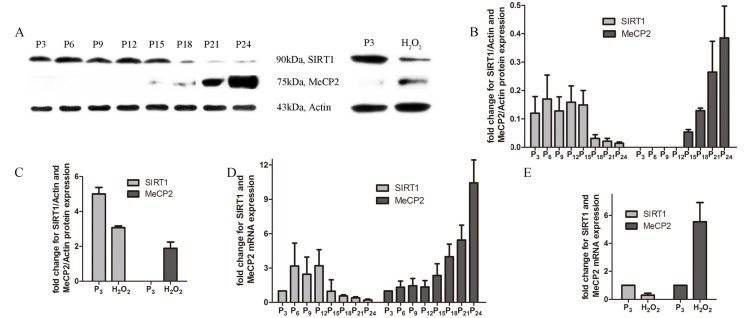
Expression of MeCP2 and SIRT1 with aging **A.**, **B.**, **C.** Protein levels of MeCP2 and SIRT1 by western blot with replicative senescence and H_2_O_2_-induced senescence. **D.**, **E.** mRNA levels of MeCP2 and SIRT1 by RT-PCR with replicative senescence and H_2_O_2_-induced senescence (experiments were repeated three times, and the same trend resulted).

Expression levels of MeCP2 and SIRT1 were examined in different EPCs. With repeated subculture, MeCP2 protein and mRNA levels increased, whereas SIRT1 levels declined. In contrast to untreated EPCs, we found increased MeCP2 protein and mRNA levels and decreased SIRT1 levels in H_2_O_2_ co-cultured EPCs (Figure 5A-5E).

### MeCP2 mediated-EPC dysfunction

**Figure 6:**
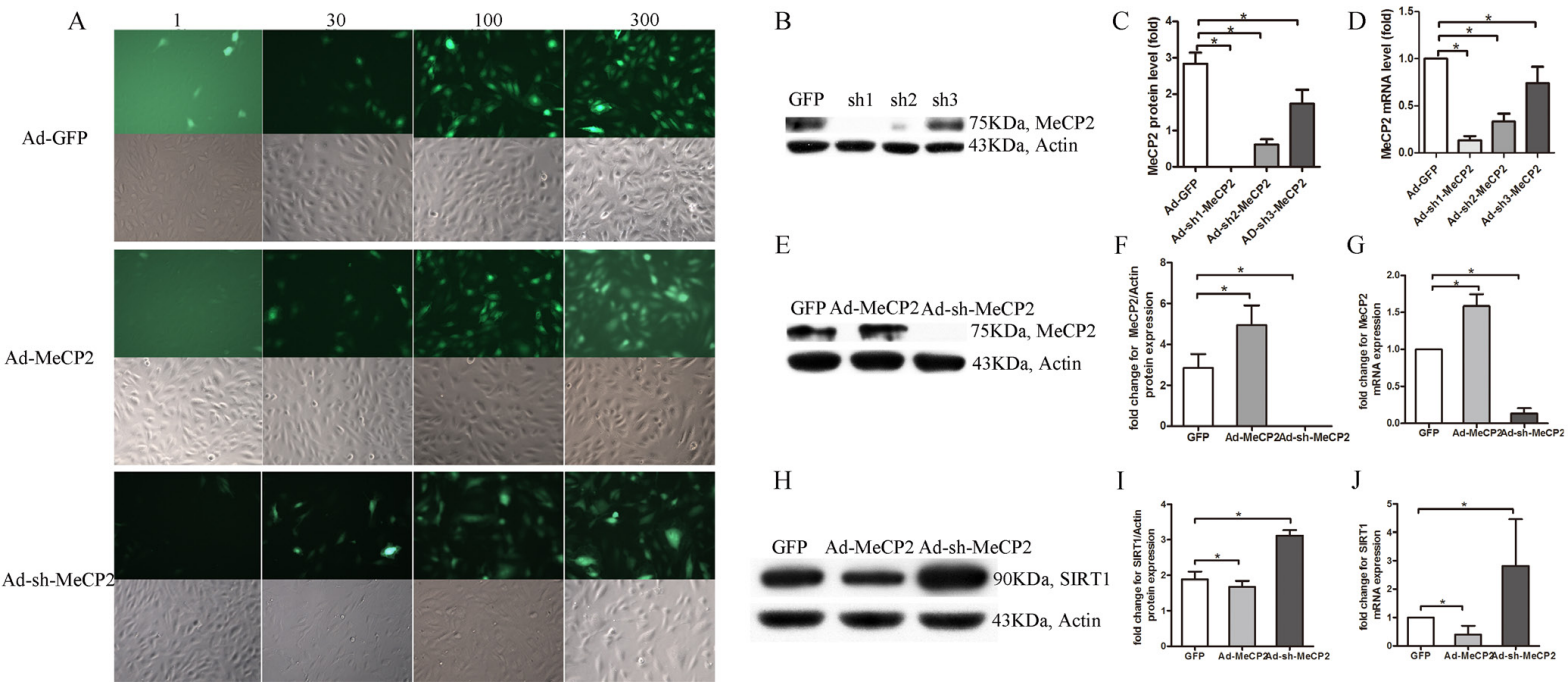
EPCs transfected with Ad-MeCP2 or Ad-sh-MeCP2 **A.** Fluorescence images of EPCs transfected with Ad-MeCP2 or Ad-sh-MeCP2 at different MOIs. **B.**, **C.**, **E.**, **F.** The effects of Ad-MeCP2 (MOI 100) and Ad-sh-MeCP2 (MOI 100) on the expression of MeCP2 were confirmed by western blot. **D.**, **G.** The effects of Ad-MeCP2 (MOI 100) and Ad-sh-MeCP2 (MOI 100) on the expression of MeCP2 were confirmed by RT-PCR analysis. **H.**, **I.** SIRT1 expression of EPCs transfected with Ad-MeCP2 or Ad-sh-MeCP2 was examined by western blot. **J.** SIRT1 expression of EPCs transfected with Ad-MeCP2 or Ad-sh-MeCP2 was examined by RT-PCR analysis. (n=3, *P<0.05 vs. GFP).

EPCs were transfected with Ad-MeCP2, Ad-sh-MeCP2, or Ad-GFP. The percentage of bright fluorescent spots increased with increasing MOI (Figure 6A), and the expression of MeCP2 affected by Ad-MeCP2 and Ad-sh-MeCP2 was confirmed by WB and RT-PCR analysis (Figure 6B-6G). MOI 100 was selected for subsequent studies.

**Figure 7:**
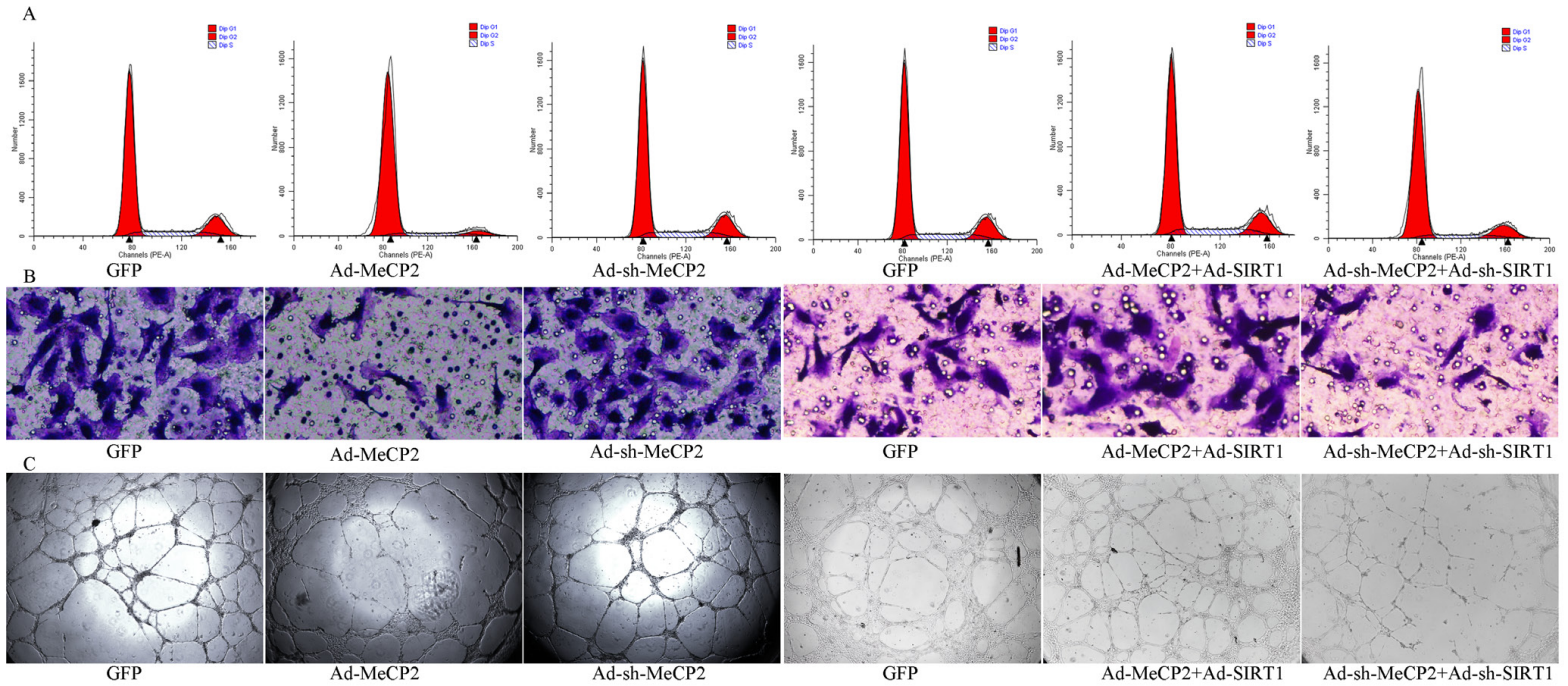
Functional alterations with overexpression or silencing by co-transfection of MeCP2 and SIRT1 **A.** Proliferation by flow cytometry (proliferation index (PI) was used to compare the proliferation ability, PI=(S+G2M)/(G0/1+S+G2M)). **B.** Migration ability by Transwell assay (X100, the number of dyed purple cells in three randomly microscopic fields were used to access migration ability). **C.** Tube formation of EPCs (X40, total loops were used to evaluate the tube formation ability). (three independent experiments were repeated, and the same result were done).

From the functional assay, we found that the proliferation of EPCs was significantly reduced by MeCP2 overexpression and elevated by MeCP2 knockdown (Figure 7A). The amount of cell migration in GFP-transduced EPCs was significantly greater than that of Ad-MeCP2, and less than that of Ad-sh-MeCP2 (Figure 7B). The tube formation capacity of EPCs was lowered by MeCP2 overexpression and increased by MeCP2 knockdown (Figure 7C).

### MeCP2 down-regulates SIRT1 levels

Because MeCP2 mediates EPC impairment and our previous work confirmed that SIRT1 improves EPC function, we investigated the expression of SIRT1 with different levels of MeCP2 in EPCs. Transfection of EPCs with Ad-MeCP2 significantly reduced SIRT1 protein and mRNA levels, whereas Ad-sh-MeCP2 transfection increased SIRT1 levels (Figure 6H-6J).

### SIRT1 reversed dysfunction in EPCs induced by MeCP2

From WB and RT-PCR assays, we obtained evidence that MeCP2 down-regulated SIRT1 expression; then, we confirmed that MeCP2 inhibited EPCs function through SIRT1. We observed the function alteration through co-transfection (overexpression of MeCP2 and SIRT1 simultaneously; MeCP2 and SIRT1silence simultaneously).

The reduced proliferation induced by MeCP2 was restored by SIRT1. The detrimental effect of MeCP2 on EPCs migration was considerably attenuated by SIRT1. MeCP2 mediated repression of EPC tube formation was rescued by SIRT1 (Figure 7).

## DISCUSSION

Human EPC-based therapies appear to be a promising approach to clinical cell-based therapy not only because of their contribution to neovasculogenesis in cardiac vascular diseases such as ischemic hindlimbs and myocardial infarction [31-35], but also because of their prevention of deleterious remodeling and improvement of recovery [36]. Therefore, the number and functional activity of EPCs are positively linked to their effects during treatment. Senescence and other risk factors, which alter both EPC morphology and function, greatly hamper clinical applications.

EPCs have been cultured from human cord blood and through replicative subculture until P25, where cells enter a state of growth arrest through a series of senescent markers; this stage was identified as replicative senescence in our study. Cells that were co-incubated with 20µM H_2_O_2_ for 24 h and replaced with new EGM-2 for 3 days of re-culture also exhibited the same state as P25 and were identified as being in stress-induced premature cellular senescence [37]. It has been proposed that senescence is driven by telomere attrition and triggers the induction of tumor suppressors including p16 and p53 [38]. P16 was found to be highly expressed in senescent cells [39]. In this study, we also confirmed that senescent cells had lower telomere activity and higher p16, and p53 mRNA levels. It was concluded based on the production of a ROS marker that H_2_O_2_ induced widespread oxidative damage, eventually resulting in aging. We also confirmed EPC functional impairment with senescence.

It is assumed that intrinsic and extrinsic factors influence the aging process through epigenetic mechanisms [40]. Many studies have proven the epigenetic mechanisms of genome regulation during aging, specifically DNA methylation and histone modifications. Age-associated hyper-methylation has been shown to occur [41]. SIRT1 and MeCP2 are two critical epigenetic regulators involved in DNA methylation and histone modifications in aging. Several studies have shown SIRT1 mediates protection against cellular senescence. MeCP2, a reader of DNA methylation signals, was directly proportional to the DNA methylation level. MeCP2 overexpression in EPCs led to higher DNA methylation. We found more MeCP2 expression and less SIRT1 expression in EPCs with senescence, which demonstrated that there was hyper-methylation with senescence.

SIRT1 is a well-known accepted longevity gene protein, and it can prolong the lifespan of a variety of organisms. It was concluded from our previous work that SIRT1 could improve the functional activities of EPCs, and decreased expression of SIRT1 was likely an important epigenetic regulation resulting in EPC impairment. In this study we found that MeCP2 mediated EPC impairment. In addition, MeCP2 functioned as a down-regulator of SIRT1 expression. After co-transfection with the adenoviral vectors overexpressing or silencing MeCP2 and SIRT1, the function of EPCs exhibited the opposite effect of independent MeCP2 alterations. All these results demonstrate that MeCP2 mediates EPC dysfunction by repressing SIRT1. The mechanism by which it inhibits the transcription of SIRT1 will be reported in due course.

Our study has several limitations. The ageing process is slow and complicated, many regulators and signaling pathways are involved in it rather than a single factor. We identified MeCP2 and SIRT1, but there may be other regulators. MeCP2 may not inhibit SIRT1 directly but through other factors. All these possibilities need further study.

## MATERIALS AND METHODS

### EPC isolation and culture

This work was approved by the Ethics Committee of Xinhua Hospital Affiliated to Shanghai Jiao Tong University, and consent from donors was received. Human cord blood mononuclear cells (MNCs) isolated by density gradient centrifugation with Histopaque-1077 (Sigma) were suspended in complete EGM-2 medium (Lonza Clonetics) and then seeded on fibronectin (Gibco) pre-coated 24-well plates, approximately 9 × 10^6^ cells were collected from 20 ml of cord blood, and 1.5 × 10^6^ cells were plated per well. Medium was changed every 3 days until the first passage. For identification of EPCs, cells were stained with antibodies against the endothelial markers CD34 (Corporation) and VEGFR-2 (Santa Cruz Biotechnology) as well as progenitor marker CD133 (Corporation), and Flow cytometry analysis was used, dual staining was used for acetylated low density lipoprotein (Dil-ac-LDL, Molecular Probes) uptake and UEA-1 (Sigma) binding.

### Western blot analysis

Cell proteins were extracted by using RIPA buffer. Protein concentration was measured with the BCA method. Approximately 30 μg of protein from each sample was loaded on 8% SDS-PAGE gels and run at 80V constant voltage. A constant current of 300 mA was used for transblotting. Blots were probed with rabbit anti-MeCP2 antibodies (1:1,000, Abcam) and anti-SIRT1 antibodies (1:2,000, Abcam) overnight at 4°C. After washing three times, blots were then incubated with goat anti-rabbit secondary antibody (1:1,000) at room temperature for 2 h. Then, chemiluminescence was used to visualize protein bands. The housekeeping gene β-actin (anti-β-actin antibody, 1:1000, Abcam) was used as internal control. The protein abundance of MeCP2 and SIRT1 was calculated based on the comparative gray value to β-actin.

### Real-time PCR

Total RNA was isolated from EPCs by using the Trizol method. Real-time polymerase chain reaction (RT-PCR) was performed using an ABI 7500 Real-Time PCR System (Applied Biosystems) with SYBR^®^ Premix Ex Taq™ II (TaKaRa). The house keeping gene GAPDH was used as an internal reference. Sequences of the primers were as follows: SIRT1: forward, 5’-GCTTCTTGGAGACTGTGA TGTC-3’ and reverse, 5’-TGTTCGTGGAGGTTTTTCAG-3’; MECP2: forward, 5’- CGAAAAGGTAGGCGACACAT-3’ and reverse, 5’-TGGGAGATTTGGGCTTCTTA-3’; GAPDH: forward, 5’-CCATGGAGAAGGCTGGGG-3’ and reverse, 5’-CAAAGTTGTCATGGATGACC-3’. The mRNA abundance of MeCP2 and SIRT1 was normalized to that of GAPDH and calculated based on the comparative 2^-ΔΔCt^ method.

### Cell viability and proliferation assay

The Cell Counting Kit-8 (CCK-8, Dojindo) was used to analyze the effects of H_2_O_2_ on EPC viability according to the manufacturer’s protocols. To determine the most suitable concentration, different concentrations (1 µM, 10 µM, 20 µM, 40 µM, 80 µM, 100 µM, 150 µM, 200 µM, 300 µM) of H_2_O_2_ were incubated with 1 × 10^4^ cells for 24 h in 96-well plates, and CCK-8 was added (10 µl/well), the incubation was then continued for 2 h. Absorbance was measured using a microplate reader at 450 nm to determine the number of vital cells in each well.

### Flow cytometry analysis

FxCycle PI/RNase Staining Solution (Invitrogen) was used to test the cell cycle according to the manufacturer’s instructions. Cells at 1 × 10^5^ were fixed in precooled 75% alcohol at 4°C overnight. After washing with PBS, PI/RNase Staining Solution was added and incubated for 30 min at room temperature in the dark. Cell cytometry analysis was performed using a BD FACSCanto II(BD Biosciences) and FlowJo version 9.3.2.

### Migration assay

Transwell plates (Corning) were used to observe the cell migration according to our protocol. A total of 600 µl of complete EGM-2 was added to the bottom chamber of the Transwell plate, and 2 × 10^4^ cells were suspended in 200 µl of EBM-2 alone in the top chamber of the well. After incubation at 37°C for 12 h, transmigrated cells were fixed in 4% paraformaldehyde, and stained with Crystal Violet staining solution, and then washed with PBS for three times, three random microscopic fields were selected to enumerate the cells.

### Matrigel angiogenesis assay

µ-Slide Angiogenesis dishes (ibidi, Germany) were used according to the manufacturer’s instructions. 10 μl of Matrigel™ (BD Biosciences) was coated in each μ-slide angiogenesis well and incubated at 37^°^C for 30 min, EPCs were seeded on the Matrigel coated μ-slide angiogenesis well plate at the density of 1 × 10^4^ cells per well. 50 μl of complete EGM-2 was used as medium for the assay. Images were captured 8 h after seeding, and total loops were measured.

### SA-β-Gal assay

SA-β-Gal staining (Cell Signaling Technology, America) was performed according to the manufacturer’s instructions, 1 × 10^5^ cells were rinsed three times with PBS, fixed with 0.5 ml of fixative solution for 20 min at room temperature, and rinsed again with PBS. Then the cells were incubated with the staining solution overnight at 37°C, and senescent cells were identified as bluish green-stained cells under a phase-contrast microscope.

### Detection of intracellular oxygen radicals

The Reactive Oxygen Species Assay Kit (Sigma) was used to measure cellular reactive oxygen species (ROS). EPCs were seeded onto 6-well-plates with a density of 1 × 10^4^ cells per well one day prior to treatment. They were washed with PBS and incubated with 10 µmol/L 2’,7’-dichlorofluorescin diacetate (DCFH-DA) with the medium at 37°C with 5% CO2 for 30 min, and then were washed 3 times with EBM-2 alone. Absorbance was measured using fluorescence enzyme labeling at excitation and emission wavelengths of 485 and 535 nm respectively.

### Telomerase ELISA assay

Telomerase(TE) was detected by using a TE ELISA kit (mlbio, China) according to the manufacturer’s instructions. A standard curve was made with the absorbance of different concentrations of reference standard as the abscissa. The TE concentration of every sample was calculated according to the regression equation.

### Adenovirus transfection

Adenoviral vectors containing green fluorescent protein (GFP) and harboring wild-type MeCP2 (Ad-MeCP2), SIRT1 (Ad-SIRT1), and short hairpin RNA MeCP2 (Ad-sh-MeCP2) and SIRT1 (Ad-sh-SIRT1) were purchased from Han Heng (Shanghai, China). Adenoviral vectors carrying only GFP(Ad-GFP) were used as the control. EPCs were transfected with Ad-GFP, Ad-MeCP2, or Ad-sh-MeCP2 at various multiplicities of infection (MOI) from 1 to 300 and incubated for 2 h. Afterward, the media was exchanged for fresh EGM-2, and the transfected EPCs were cultured for an additional 44 h. The MOI of Ad-SIRT1 and Ad-sh-SIRT1 used in this work was previously described [41]. The effects of Ad-MeCP2 and Ad-sh-MeCP2 on the expression of MeCP2 were detected by fluorescence microscopy, western blot and RT-PCR analyses.

### Statistical analysis

The results are expressed as the mean±SE. Comparisons between two groups were analyzed by two independent sample t-test. A probability value of *P*<0.05 was considered to be statistically significant. All experiments were independently performed at least in triplicate. All analyses were performed with SPSS 13.0 software.
